# Low glomerular number at birth can lead to the development of chronic kidney disease

**DOI:** 10.3389/fendo.2023.1120801

**Published:** 2023-01-26

**Authors:** Shohei Fukunaga, Yuki Fujita

**Affiliations:** ^1^ Division of Nephrology, Shimane University Hospital, Izumo, Shimane, Japan; ^2^ Department of Developmental Biology, Shimane University Faculty of Medicine, Izumo, Shimane, Japan

**Keywords:** glomerular number, chronic kidney disease, maternal malnutrition, low birth weight, nephron development

## Abstract

Chronic kidney disease (CKD) prevalence is increasing worldwide, and reducing the number of patients with CKD is of utmost importance. The environment during the fetal, perinatal, and early childhood stages may influence CKD development (developmental origins of health and disease). Under conditions of maternal malnutrition, the glomerular number of infants reduces, and the risk of developing CKD may increase. Nephron progenitor cells and ureteric buds interact with each other to form glomeruli at the tip of the ureteric bud. Thus, the number of glomeruli is determined by the number of ureteric bud branches, which are reportedly decreased due to maternal malnutrition, in turn reducing the glomerular number. Four possible mechanisms can explain the low glomerular number resulting from maternal malnutrition: 1) suppression of *c-Ret* expression, 2) suppression of nephron formation by renin-angiotensin-aldosterone system inhibition, 3) exposure to excess glucocorticoids, and 4) promotion of apoptosis. Additionally, nephron formation does not continue after birth in humans. Therefore, a low glomerular number at birth is a lifelong burden on the glomeruli and increases the risk of developing CKD. Therefore, it is important to maintain the glomerular number at birth. Accurate glomerular counts are essential for conducting studies on the glomerular number. The dissector/fractionator method is the gold standard; however, it can only be performed at some institutions. Recently, methods have been developed to measure the glomerular number by combining computed tomography and pathological examination and measure the glomerular count using magnetic resonance imaging. Models of decreased and increased glomerular numbers have been developed. Moreover, research regarding the causes of decreased glomerular number and its relationship with development of lifestyle-related diseases and renal dysfunction has significantly progressed, furthering our understanding of the importance of glomerular number.

## 1 Introduction

Chronic kidney disease (CKD) is becoming more prevalent worldwide; a prevalence rate of 9.1% was reported in 2017, with an increase of 29.3% since 1990 (1). In addition, approximately 2.6 million patients received renal replacement therapy in 2010, with 5.4 million expected to require it by 2030 (2). To prevent this, CKD onset and progression must be controlled. Furthermore, low-birth-weight infants have low glomerular numbers (3, 4) and are at a higher risk of developing CKD (5), indicating a link between the glomerular number and CKD development risk. This review focuses on the relationship between the glomerular number and renal function, causes of glomerular number decline, and interventions currently under investigation for glomerular number maintenance.

## 2 Development of glomerulus

The nephrons of the kidney originate from the branching of the ureteric bud, which interacts with the nephron progenitor cells that epithelialize to form the proximal tubules. Then, capillaries are induced to form glomeruli at the ends of branched tubular buds; therefore, the glomerular number is determined by the number of ureteric bud branches. In rats, the formation of nephrons begins on embryonic day (E) 12 and continues for 8–11 postnatal days (6). In mice, it begins on E 11 and continues for 7 postnatal days (7). Nephrogenesis is ongoing at birth in rodents. In humans, nephrons form between 9 and 34–36 weeks of gestation, and glomerulogenesis ceases at birth. However, in preterm infants, nephrons continue to form until 40 postnatal days (8), after which the number of glomeruli does not increase. Therefore, if the number is low at birth, the glomeruli will be overtaxed throughout life.

## 3 Relationship between glomerular number and renal function

**Figure 1:**
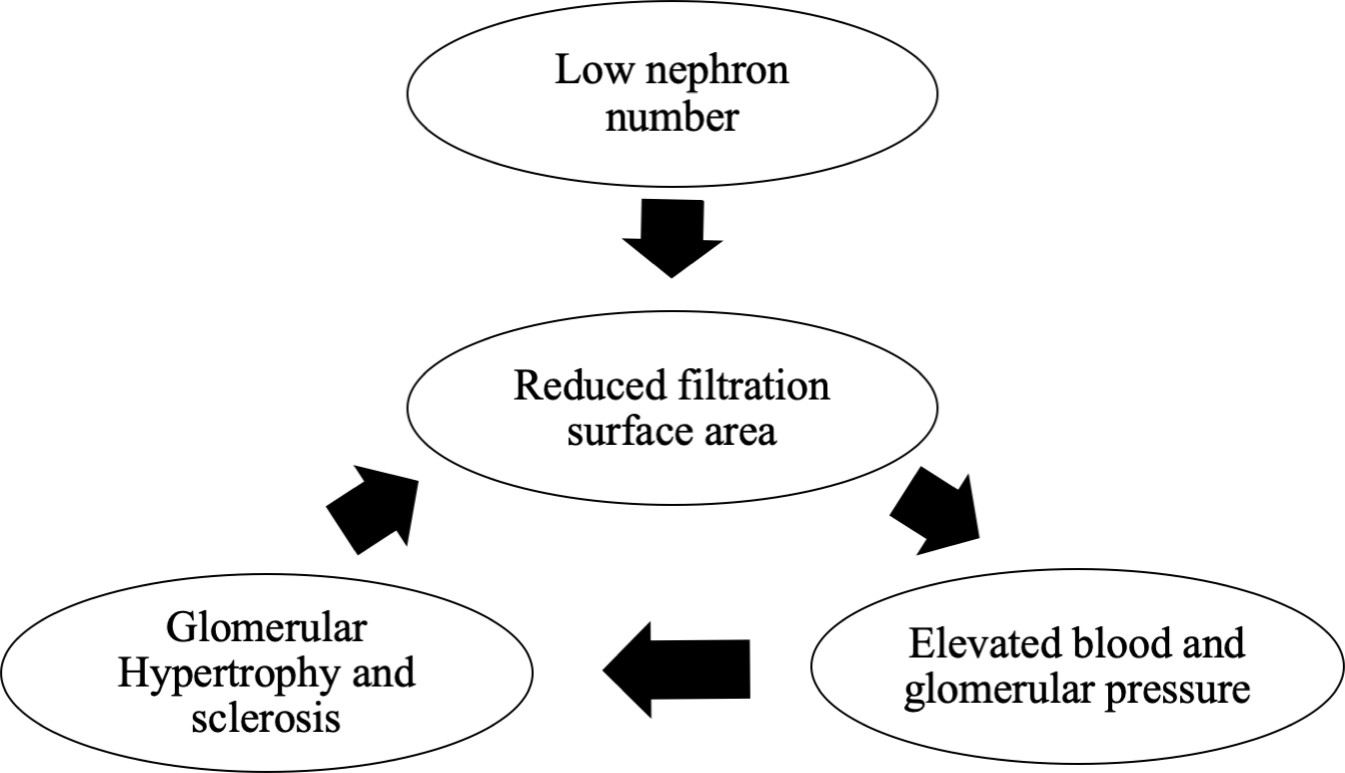
Brenner’s hypothesis.

Brenner et al., in their study in 1989, proposed that “essential hypertension is caused by a decrease in the number of glomeruli and nephrons, which results from an undesirable prenatal intrauterine environment (maternal malnutrition, stress, and chemical exposure), genetic factors, and premature birth. Low glomerular number leads to renal glomerular hypertrophy, and eventually to hypertension, CKD, nephrosclerosis, and renal failure” (Brenner’s hypothesis) (**Figure 1**) (9). Histological comparison of the glomerular number in autopsied men who died in a traffic accident in Germany showed a decrease in glomerular number in the hypertension group, thus supporting Brenner’s theory (10). Birth weight is a determinant of glomerular number, and a reduction in glomerular number because of low birth weight may be a risk factor for hypertension and end-stage renal failure in adulthood (11). A study of 2.18 million people born in Norway, who were observed for an average of 21 years, conducted between 1967 and 2004, reported that 526 developed end-stage renal failure with low birth weight and intrauterine fetal growth retardation as risk factors (12). Furthermore, a birth weight <2.5 kg is associated with a higher risk of end-stage renal failure than a weight of 3–3.5 kg (13), and patients with CKD show a higher incidence of low birth weight than those without CKD (14). Thus, a low glomerular number due to low birth weight is a risk factor for end-stage renal failure, and how high the glomerular number is maintained at birth may influence the future renal reserve.

## 4 Causes of decreased glomerular number at birth

While nephron formation does not occur after birth in term infants, it continues until 40 days after birth in preterm infants (8), after which the glomeruli do not increase. Thus, the total glomerular number is expected to be lower after a preterm birth. In addition, juxtamedullary nephrons may predominate over cortical nephrons, are more vulnerable to ischemia, and get easily injured due to obesity, hypertension, and diabetes (15). Thus, preterm infants may have a low glomerular number, high proportion of juxtamedullary nephrons, and may be more prone to lifestyle-related diseases.

The glomerular number reportedly demonstrates racial variation: Caucasian Americans, African Americans, Aboriginals, and Japanese have a total glomerular number of approximately 900,000 (16), 950,000 (16), 680,000 (17), and 670,000 (18), respectively (**Table 1**).

**Table 1:** Factors affecting the number of nephrons.

	Glomerular number	
	High	Low
Race (glomerular number)	Caucasian Americans (900,000), African Americans (950,000)	Aboriginals (680,000), Japanese (670,000)
Sex	Men	Women
Birth weight	>2.5 kg (normal-birth-weight)	<2.5 kg (Low-birth-weight)
Diabetes mellitus	No	Yes
Alcohol consumption	No	Yes
Smoking	No	Yes
Maternal nutritional status	No maternal malnutrition	Calories, protein, salt, vitamin A or D deficiency
Hereditary disease	No	Renal coloboma syndrome, Duane-radial ray syndrome, Axenfeld-Rieger syndrome, CHARGE syndrome, Branchio-oto-renal syndrome, Townes-Brocks syndrome, Rokitansky-Küster-Hauser syndrome

The glomerular number varies by sex, with a 12% lower glomerular number in women than in men (17, 19, 20).

Furthermore, glomerular number is positively correlated with birth weight (3, 11). For every 1.0 kg increase in birth weight, the number of glomeruli increases by 250,000. Low-birth-weight infants have an increased glomerular size compared with normal-birth-weight infants. This may indicate glomerular over-filtration due to the low glomerular number.

The glomerular number at birth is also strongly related to the maternal nutritional status; during World War II, from 1944 to 1945, some areas of the Netherlands suffered from extreme food shortages, and daily caloric intake dropped to 400–800 kcal per person, which was known as the Dutch famine. Children born to pregnant women who experienced the Dutch famine had a 3.2-fold higher incidence of microalbuminuria and 10% reduction in creatinine clearance during adulthood (21). Additionally, in mice and rats, the glomerular number has been reported to decrease with restricted protein (22, 23), caloric (24), and vitamin A (25) intake during gestation. Both high and low salt intake decrease glomerular number (26), whereas vitamin D deficiency prolongs the time taken for nephrogenesis and delays glomerular maturation (27). Animal studies have shown that nephron number decreases with deficiency of various nutrients. Among these nutrients, retinoic acid (RA), in particular, majorly influences the nephron number. In *in vitro* studies, the addition of RA to the culture medium increases the glomerular number (28). In rats, protein restriction decreases nephron number, but protein restriction and administration of RA have been reported to improve nephron number to levels comparable to those of controls (29). In contrast, postnatal administration of RA to preterm baboons does not affect nephron number (30). This may be because nephrogenesis ceases after birth in mammals. Therefore, it is important to ensure that RA is not deficient during gestation.

Four mechanisms have been postulated for the reduction of glomerular number due to low nutritional exposure:

1. Suppression of *c-Ret* expression

**Figure 2:**
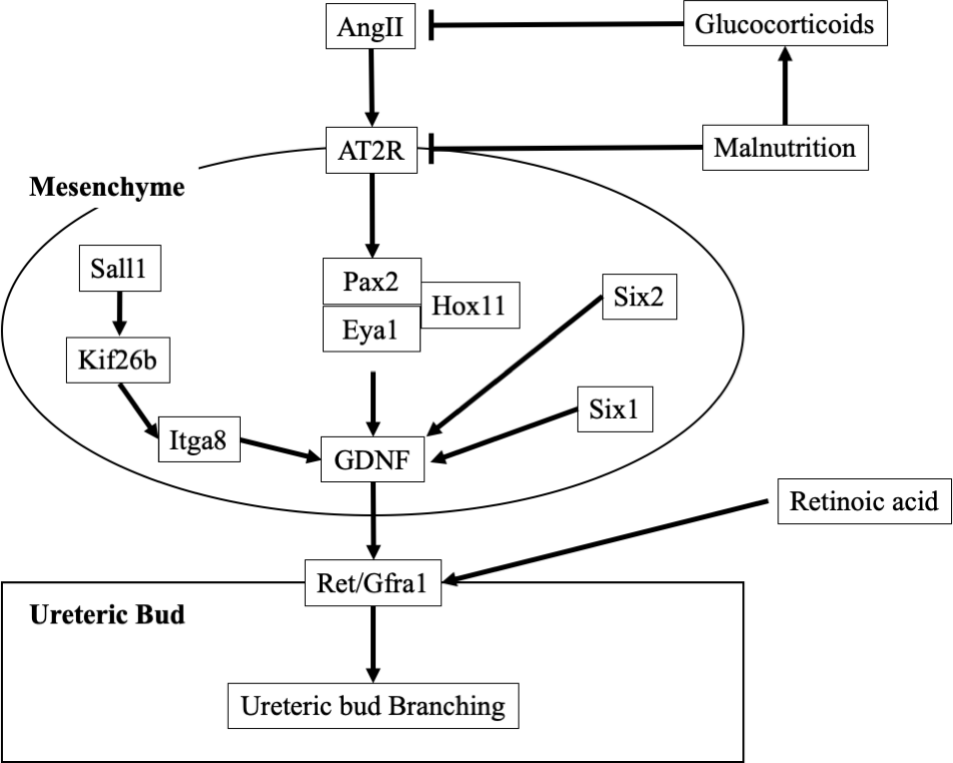
Molecular mechanisms of ureteric bud branching. AngII, Angiotensin II; AT2R, AngII type 2 receptor; Eya1, Eyes absent homolog 1; GDNF, Glial-cell-line-derived neurotrophic factor; Gfra1, GDNF family receptor α1; Hox11, Hombox gene 11; Itga, Integrin subunit aplpha; Kif, kinesin superfamily proteins; Pax2, Paired box gene; Ret, ret proto-oncogene; Sall, sal-like; Six, Sine oculis-related homeobox.

Glial-cell-line-derived neurotrophic factor (*GDNF*), which is secreted from the mesenchyme, acts on the Wolffian ducts to germinate and elongate ureteric buds (31) (**Figure 2**). The GDNF receptor molecule ret proto-oncogene (*Ret*) and its co-receptor GDNF family receptor α1 (*Gfra1*) are expressed in the ureteric bud, and *GDNF* secreted in the mesenchyme transmits signals to the ureteric bud *via Ret*. *GDNF-Ret/Gfra1* signaling is required for ureteric bud germination and elongation. Vitamin A-dependent *c-Ret* expression and nephron formation (32): Vitamin A deficiency suppresses nephron formation, and nephrons form in a vitamin A concentration-dependent manner in culture mediums, which is related to the *c-Ret* expression (33). RA increases *c-Ret* gene expression independent of the *GDNF* gene expression, suggesting that RA may have a direct effect on *c-Ret* expression.

2. Suppression of nephron formation by renin-angiotensin-aldosterone system inhibition

The renin angiotensin aldosterone system (RAAS) seems to be critically important in renal organogenesis. Hombox gene 11 (*Hox11*)/Eyes absent homolog 1 (*Eya1*)/Paired box gene 2 (*Pax2*) form a complex that directly regulates *GDNF* expression and promotes ureteric bud branching (34). Angiotensin II (AngII) upregulates *Pax2* gene expression *via* the AngII type 2 receptor (AT2R). Additionally, AT1R and AT2R expressions are strongly suppressed in a hypotrophic *in utero* environment (35). Therefore, suppression of the renin-angiotensin-aldosterone system in a low-nutritional environment suppresses renal glomerulogenesis. Furthermore, it has been reported that after glomerulogenesis, if the mother is diabetic (36) or obese (37), RAAS activation causes NF-κB activation, which enhances podocyte apoptosis, leading to a decrease in nephron number.

3. Exposure to excessive glucocorticoids

When pregnant women are administered high doses of glucocorticoids, infants show decreased birth weight, decreased renal weight, hypertension, proteinuria, decreased sodium excretion capacity, and increased tissue sodium content. When pregnant rats are fed a protein-restricted diet, the placenta suppresses the release of the enzyme 11β-hydroxysteroid dehydrogenase type 2, which deactivates glucocorticoids. Therefore, glucocorticoid deactivation is suppressed, and more active glucocorticoids are transferred to the fetal side. In addition, suppression of 11β-hydroxysteroid dehydrogenase type 2 expression and increased glucocorticoid receptor expression occur in the kidney (38). When the kidney is exposed to excess glucocorticoids, the expression of genes associated with the renin-angiotensin-aldosterone lineage is suppressed. In particular, AT2R-mediated *Pax2* expression is suppressed, and glomerulogenesis is inhibited (39).

4. Increased apoptosis

In low nutritional states, the glomeruli undergo apoptosis and decrease in number. Moreover, under hypotrophic conditions, the expression of DNA methyltransferases is suppressed. Hypomethylation results in *p53* overexpression, which increases the expression of apoptotic Bax and Caspase-3 and suppresses that of cell growth factors, including Bcl-2 and IGF-1. Both these phenomena are assumed to enhance apoptosis, resulting in glomerular apoptosis, thereby decreasing the glomerular number.

Recently, maternal and paternal effects on epigenetic mechanisms have been reported. A chronic high-fat diet administered to father rats caused diabetes mellitus in the next generation of female rat pups due to pancreatic beta cell dysfunction (40). Therefore, paternal lifestyle may affect the glomerular number of the offspring, and further studies are warranted.

In addition to maternal malnutrition, other complications, such as diabetes mellitus (36), alcohol consumption (41, 42), and smoking (43, 44), have also been reported to decrease the glomerular number at birth.

Hereditary disease can also cause abnormal kidney development, resulting in low nephron number. However, the identification of the causative gene is difficult, because kidney development is intricately related to a large number of genes (**Figure 2**). The identification rate of causative genes for congenital anomalies of the kidney and urinary tract (CAKUT) is reported to be low, with only 6.3% of CAKUT causes identified (45). Among the most frequently reported causative genes for CAKUT is an abnormality in the *Pax2* gene, which causes renal coloboma syndrome. This disease is associated with renal hypoplasia and ocular manifestation (loss of some normal ocular tissue) (46). Diseases similar to renal coloboma syndrome include Duane-radial ray syndrome (abnormal gene: sal-like (*Sall*) 4), Alagille syndrome (abnormal genes: *Jagged 1*, *NOTCH1*), Axenfeld-Rieger syndrome (abnormal gene: *Forkhead Box C1*), and CHARGE syndrome (abnormal genes: Chromodomain Helicase DNA Binding Protein 7 [*CHD7*], Semaphorin-3E [*SEMA3E*]). Branchio-oto-renal (BOR) syndrome is caused by mutation in the *EYA1*, Sine oculis-related homeobox (*Six*) *1*, *Sall1*, and *Six 5* genes (47). This disease has three main features: gill-derived malformation, such as cervical fistula, eustachian fistula, and external ear malformation; various types of hearing loss; and renal and urinary tract malformations (48). In addition, abnormalities in the *Sall 1* gene cause Townes-Brocks syndrome. The Mayer-Rokitansky-Küster-Hauser syndrome also causes renal hypoplasia. The disease is associated with vaginal and uterine defects. The causative gene has not been identified, but it is thought to be caused by an abnormality in a gene essential for the development of Müllerian and Wolffian ducts (49).

In addition, although the although the pathogenic mechanism is unclear, the frequency of CAKUT is approximately 4.5 times higher in children with Down syndrome than in those without it (50). Furthermore, children with Down syndrome potentially have mildly impaired renal function with an estimated glomerular filtration rate of approximately 80% of that of normal children (51).

In Alport syndrome, which is caused by other type 4 collagen abnormalities, the glomerular basement membrane is abnormal and glomerular structure cannot be maintained, leading to end-stage renal failure at a young age.

## 5 Methods to determine the glomerular count

Accurate measurement of the glomerular count is important to ascertain the glomerular number. Currently, five methods are used for glomerular counting, each with their own advantages and disadvantages (**Table 2**).

**Table 2:** Methods of determining the glomerular count.

	Accuracy	Difficulty
Acid maceration	Somewhat low	Easy
Number of glomeruli per unit area	Low	Easy
Dissector/fractionator method	High	Difficult
Renal biopsy + CT	Somewhat high	Somewhat difficult
MRI	Somewhat high	Somewhat difficult

CT, computed tomography; MRI, magnetic resonance imaging.

### 5.1 Acid maceration

Decapsulated kidneys are coarsely chopped and incubated in 6N hydrochloric acid at 37°C. The kidneys are disrupted *via* pipetting. Distilled water is added to the samples, which are then incubated overnight at 4°C. The sample is placed in a culture dish with a 2-mm grid, and the glomeruli are counted (52). This method is rapid, simple, and inexpensive. However, structural abnormalities of the glomeruli may make them susceptible to acid digestion, and counting errors may make assessment inaccurate.

### 5.2 Number of glomeruli per unit area

The number of glomeruli found in a tissue section is measured and presented as the number of glomeruli per unit area of the section. Although this method has been used in many studies, the number of glomeruli per unit area does not represent the total number of glomeruli in the kidney. The number of glomeruli observed in a tissue section is influenced not only by the number of glomeruli in the kidney but also by the size and shape of the glomeruli and thickness of the section. Larger glomeruli are more likely to appear in sections than smaller glomeruli. Therefore, the number of glomeruli per unit area alone cannot be used to evaluate the total number of glomeruli in the kidneys.

### 5.3 Dissector/fractionator method

This measurement method (53, 54) uses the Cavalieri and fractionator principles (55) and is considered the gold standard for determining the total glomerular number. The accuracy of other glomerular counting methods was validated *via* comparison with this method. However, the equipment and technology required for measurement, as well as the cost and time involved, limit the number of facilities that are equipped to use this method.

### 5.4 Renal biopsy + computed tomography

Glomerular count estimation using a renal biopsy specimen from a renal transplant donor and performing a contrast-enhanced CT before donation has been attempted (56). The glomerular density of the renal biopsy specimen was measured, and the kidney was reconstructed three-dimensionally from contrast-enhanced CT images to estimate the glomerular density and total renal cortical volume. The results of this method are similar to those obtained using the dissector/fractionator method. However, contrast-enhanced CT and renal biopsy need to be performed.

### 5.5 Magnetic resonance imaging

A method was developed to measure the glomerular count by MRI using cationic ferritin as a contrast agent. Cationized ferritin is injected intravenously to label the glomerular basement membrane of the kidney, and MRI imaging highlights the glomeruli in black. This has been reported *in vitro* in mice and rats (57) and *in vivo* in rats (58), but not in humans. An advantage of this method is that it does not require kidney specimen preparation. In addition, *in vivo* measurement may be possible; however, this method has only been reported in rats, and its safety in humans remains unclear. Furthermore, an MRI machine is required to perform this test.

## 6 Interventions to preserve nephron number at birth

### 6.1 Low-nephron number model

A low-nephron number model is necessary to examine the changes that occur in an organism when the nephron number is reduced. As aforementioned, animal models have been created with low nephron count due to maternal malnutrition. Another low-nephron number model is the hypogonadism rat model with testicular and renal hypoplasia (59). The glomerular number in this model was approximately 80% lower than the normal number. Additionally, poor ureteric bud growth, the presence of undifferentiated mesenchymal cells, and cortical non-thinning was observed, suggesting that abnormal cell proliferation results in renal hypoplasia.

Caspase-3 knockout mice also show decreased nephron numbers (60). Caspase-3 is involved in migration, proliferation, differentiation, and apoptosis. Caspase-3 knockout mice demonstrate suppressed ureteric bud branching, resulting in a low glomerular number.

The two models described above are low-nephron number models resulting from genetic abnormalities, making it difficult to control the degree of glomerular number decline.

To address that, a low-nephron number model using the Six2/iDTR model has been proposed by the author (61). This model expresses the diphtheria toxin receptor in nephron progenitor cells, which can be removed by administering diphtheria toxin to the amniotic fluid during the embryonic period. The observed kidney size and glomerular count reduction is inversely proportional to the diphtheria toxin dose, which can be employed to achieve any degree of glomerular number reduction.

### 6.2 High-nephron number models

High-nephron number models have also been developed. If a low nephron number increases the CKD risk, a high number may reduce it. One model of increased glomerular count involved transgenic mice expressing the truncated type II activin receptor (62). In this model, signaling through the activin receptor was attenuated, and the total glomerular number increased to approximately 180% of that in normal mice. However, the serum urea nitrogen, creatinine, and creatinine clearance rates were comparable to those of normal mice.

Another method reported by the authors was to boost the glomerular number by intraperitoneally administering RA during the embryonic period (63), which increased the total number of glomeruli in mouse pups by approximately 1.5-fold than that in the control group. No genetic modification was required, and the number of glomeruli could easily be increased. However, its effects on renal function remain unclear.

## 7 Clinical interventions for low nephron number

To reduce the risk of developing CKD due to low nephron number, it is important to first prevent low nephron numbers. Interventions for hereditary disease are difficult, but those for low nephron number caused by the maternal environment are possible. Pregnant women should be taught regarding proper nutrition, weight control, smoking cessation, and alcohol abstinence. In addition, management of diabetes and obesity, if present, is also important.

Once CKD progresses, it is difficult to improve renal function. Therefore, early detection of patients with low nephron numbers is crucial. Serum creatinine and cystatin C levels increase with renal dysfunction. In addition, urinary β2-microglobulin and N-acetyl-β-D-glucosaminidase are markers of tubular damage. However, all of these biomarkers are elevated after damage has occurred, and there is currently no biomarker that can detect low nephron numbers before damage has occurred.

Therefore, children at risk for low nephron numbers, such as preterm and low-birth-weight infants, should be evaluated for kidney involvement. In addition, renal evaluation is advisable, because many inherited diseases are often associated with abnormalities in renal development. Kidney size correlates with the nephron number (64), and in children, renal volume and the sum of the left and right kidney length diameters are strongly and sexually correlated with glomerular filtration rate (65). Therefore, evaluation of the kidneys should include an assessment of renal function, such as serum creatinine levels, as well as morphology using abdominal echocardiography and other techniques.

Patients with a low nephron number need to have their intraglomerular pressure lowered, as increased intraglomerular pressure causes progressive glomerulosclerosis. Renin angiotensin system inhibitors, sodium glucose cotransporter 2 inhibitors, and mineralocorticoid receptor antagonists may be effective in this regard. Dietary therapy, such as salt reduction, is also important. In addition, the first 1,000 days of life, from the fetal period to 2 years of age, are the most critical in the developmental program, and avoiding nutritional deficiencies during this period can reduce lifestyle-related diseases (CKD, hypertension, or diabetes) that may develop later in adulthood (66).

## 8 Conclusion

**Figure 3:**
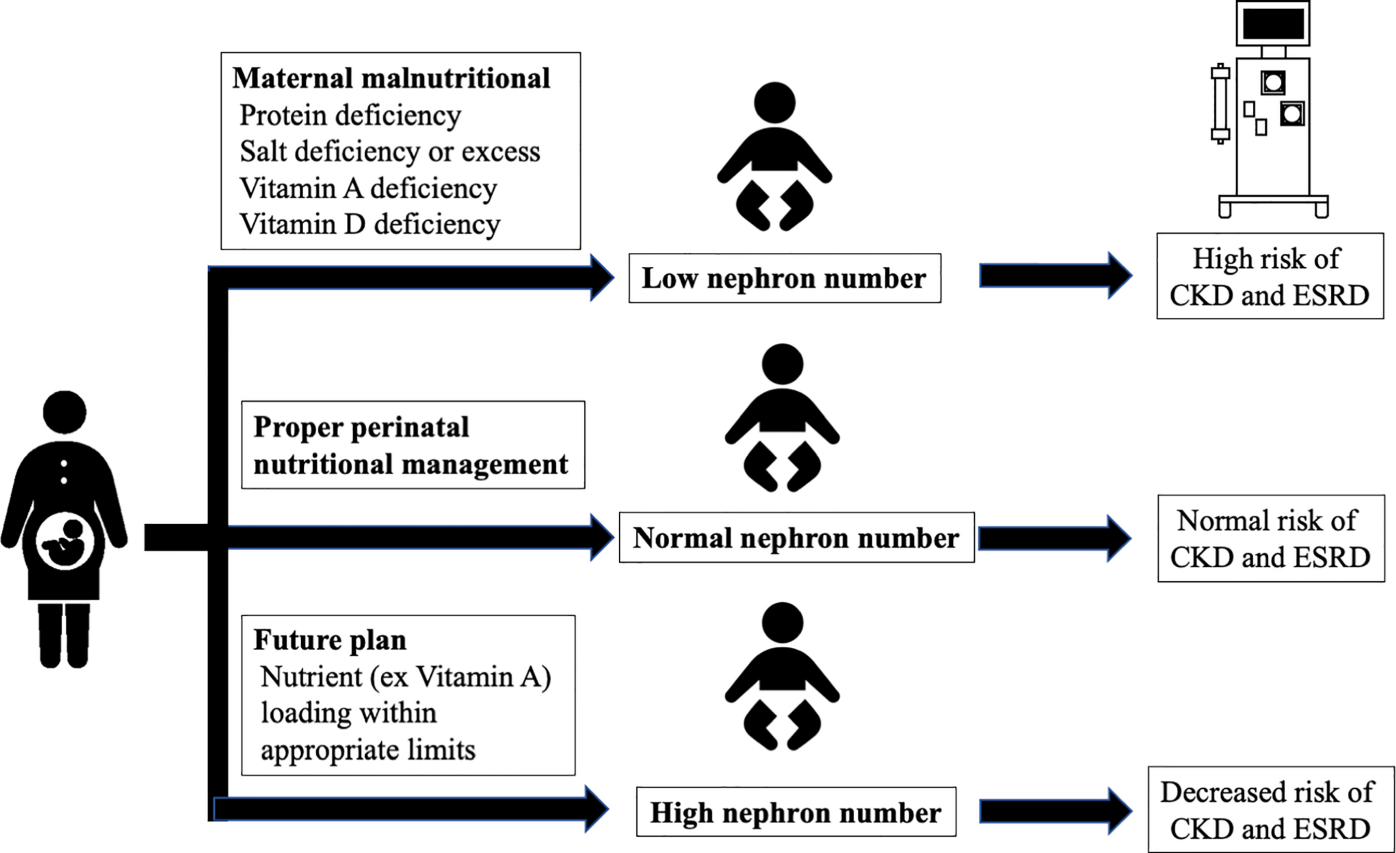
Interventions for glomerular number. CKD, chronic kidney disease; ESRD, end-stage renal disease.

An undesirable prenatal intrauterine environment, including maternal malnutrition, decreases the fetal glomerular number and increases the risk of CKD; therefore, an appropriate environment should be maintained to prevent the decrease in fetal glomerular number. Altogether, proper perinatal nutritional management and preventing the decrease of and increasing the glomerular number at birth may greatly reduce the incidence of CKD in the future (**Figure 3**).

## Author contributions

SF is the first author. Both authors contributed to the article and approved the submitted version.
